# The impacts of a GO-game (Chinese chess) intervention on Alzheimer disease in a Northeast Chinese population

**DOI:** 10.3389/fnagi.2015.00163

**Published:** 2015-08-25

**Authors:** Qiao Lin, Yunpeng Cao, Jie Gao

**Affiliations:** ^1^Department of Internal Medicine, The Fourth Affiliated Hospital of China Medical UniversityShenyang, China; ^2^Neural Department of Internal Medicine, The First Affiliated Hospital of China Medical UniversityShenyang, China; ^3^Department of Anatomy, The First Affiliated Hospital of China Medical UniversityShenyang, China

**Keywords:** Alzheimer's disease, brain-derived neurotrophic factor, GO game, Montgomery-Asberg Depression Rating Scale, RAND-36

## Abstract

A GO game can enhance mental health, but its effects on Alzheimer Disease (AD) remains unknown. To address the issue, 147 AD patients were randomly assigned into control (without GO-game intervention), Short-time GO-Game Intervention (SGGI, 1 h daily) and Long-time GO-game Intervention (LGGI, 2 h daily) groups. After 6-month follow-up, the game reduced the mean score of Montgomery-Asberg Depression Rating Scales (MADRS) of 4.72 (95% CI, 0.69 to 9.12) and Hospital Anxiety and Depression Scale (HADS) of 1.75 (95% CI, 0.17–3.68), and increased the mean score of Global Assessment of Functioning (GAF) of 4.95 (95% CI, −1.37–9.18) and RAND-36 of 4.61 (95% CI, −2.75–11.32) (*P* < 0.05 via controls). A GO-game intervention improved 9 of 11 items of KICA-dep (Kimberley Indigenous Cognitive Assessment of Depression). Meanwhile, serum levels of brain derived neurotrophic factor (BDNF) were higher in SGGI and LGGI groups (24.02 ± 7.16 and 28.88 ± 4.12 ng/ml respectively, *P* = 0.051) than those in controls (17.28 ± 7.75 ng/ml) (*P* < 0.001). The serum levels of BDNF showed a negative relation with MADRS and a positive relation with RAND-36 (*P* < 0.01). A GO-game intervention ameliorates AD manifestations by up-regulating BDNF levels.

## Introduction

Alzheimer's disease (AD) is one leading cause of dementia and affects more than 35 million people in the world (Gregori et al., 2015; Hamilton et al., 2015). AD is a kind of neurodegenerative disorder and mainly characterized by extracellular senile plaques and intracellular neurofibrillary tangles. The high incidence and financial global burden of AD manifestations greatly reduce the quality of elderly life. However, the etiology of AD is complex and medicine therapy is still the mainstay for AD patients. Cholinesterase inhibitor and memantine (1-amino-3,5-dimethyl-adamantane) are often used for the conventional therapy of AD patients (Ahn et al., 2014; Dysken et al., 2014; Evans et al., 2014). However, all the medicine has significant side effects. Cholinesterase inhibitors cause the side effects, such as bradycardia (Leikin et al., 2014), hypotension (van Beek et al., 2010), and low intraocular pressure (Sawada et al., 1971). Memantine causes common adverse effects, such as confusion, dizziness, drowsiness, headache, insomnia and hallucinations, and some less common adverse effects including vomiting, anxiety, hypertonia, cystitis and libido (Rossom et al., 2004; Jain et al., 2008). Furthermore, it can induce reversible neurological impairment in sclerosis (an inflammatory disease in the brain and spinal cord) patients, resulting in the halt of a clinical trial (Villoslada et al., 2009). High cost of the medicine and serious unwanted adverse effects, have limited its utilization. Therefore, it is highly demanded to explore low-cost non-pharmaceutical method for AD patients.

Brain exercises are widely known approaches to prevent dementia, increase neurogenesis and protect neurons (Gatz, 2005). Physicians often advise the elderly to engage in a mentally challenging activity to reduce dementia risk. The elderly spend time solving crossword puzzles, anagrams and figural logic puzzles, and may feel well if the activities are both challenging and successfully completed. More education has been found to be related to a lower incidence of AD. Typically, the risk of AD is 2–4 times higher in the persons with less education than those with more education (Qiu et al., 2001). On the other hand, the elderly participate in leisure activities, especially for mental stimulation, will also have a lower incidence of AD (Provencher et al., 2009). In addition, longitudinal studies have showed older adults have less dementia if they participate in intellectually challenging activity (Hultsch et al., 1999).

The GO game, a kind of Chinese chesses, has been practiced as a brain activity for more than 5000 years. Playing the game involves many aspects of cognition and improves mental health (Kim et al., 2014). A GO game involves the changes associated with many cognitive functions, including learning, abstract reasoning, and self-control, which facilitate cognitive behavioral therapy (Lee et al., 2010). However, the functional role of a GO game in AD patients remains elusive. Furthermore, brain derived neurotrophic factor (BDNF) is often deficient in AD patients or animal AD models (Voineskos et al., 2011; Lee et al., 2012; Boiocchi et al., 2013). BDNF protects cerebral cortical neurons against beta-amyloid 25–35 (the major AD toxic peptide) and inhibits beta-amyloid 25–35 aggregation in the brain (Xiao et al., 2010; Zhang et al., 2012). A GO game may ameliorate AD patients by affecting the levels of BDNF since BDNF plays a critical role in learning and memory functions (Fan et al., 2014). Thus, we explored the functions of a GO game in AD patients and measured BDNF levels in AD patients before and after the study.

## Materials and methods

### Participants

All the protocols were approved by the Institutional Ethics Committee from the Fourth Affiliated Hospital of China Medical University and conducted in accordance with the Declaration of Helsinki (Palacios, 2013). All subjects were Han Chinese from Shenyang city (China). An informed consent was obtained either from each subject or from his or her guardian. All AD patients were diagnosed with NINCDS-ADRDA (National Institute of Neurological and Communicative Disorders and Stroke and the Alzheimer's Disease and Related Disorders Association) criteria according to a previous report (Tamaoka, 2011). All familial cases of AD were excluded in this study. The patients were excluded if they met any of the following criteria: (1) a history of suicidal behavior; (2) substance abuse; (3) verbal communication difficulty; (4) a family history of AD; (5) brain injury or other brain disorders; (6) disliking playing a GO game. From July 7th, 2014 to January 8th, 2015, a total of 147 AD patients were recruited at the Fourth Affiliated Hospital of China Medical University (Shenyang, China).

### Study design

**Figure 1:**
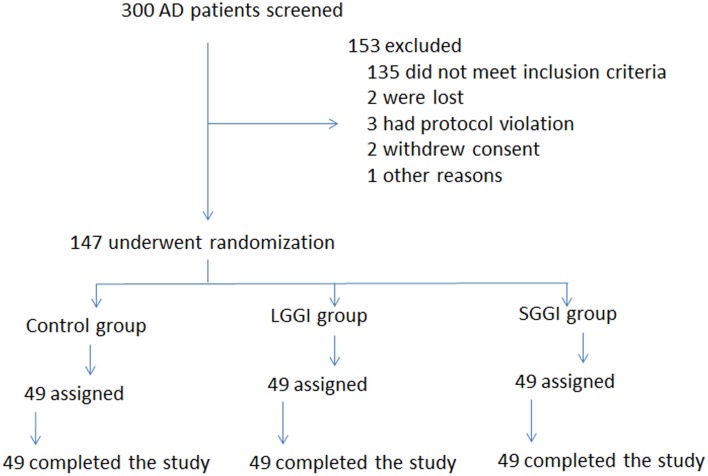
**The flowchart of the study**. LGGI, a long-time GO game intervention. SGGI, a short-time GO game intervention. Control group, without a GO game intervention.

After scanning all AD patients, 147 patients were eligible to enter designated 6-month follow-up (Figure 1). A randomized and controlled trial was conducted using three groups: control (without GO-game intervention, *n* = 49), short-time GO-game intervention (SGGI, 1 h daily, *n* = 49) and long-time GO-game intervention (LGGI, 2 h daily, *n* = 49) groups. The three groups have the similar gender ratio, age, BMI (body mass index, the ratio of weight (kg) to height in meters squared), years of education, alcohol drinking, cigarette smoking, and diabetes. Each of them has a spouse and there is a good mutual relation. Among the AD patients, some subjects have complex disorders such as comorbid diabetes, depression, and overeating (Brownley et al., 2015).

**Figure 2:**
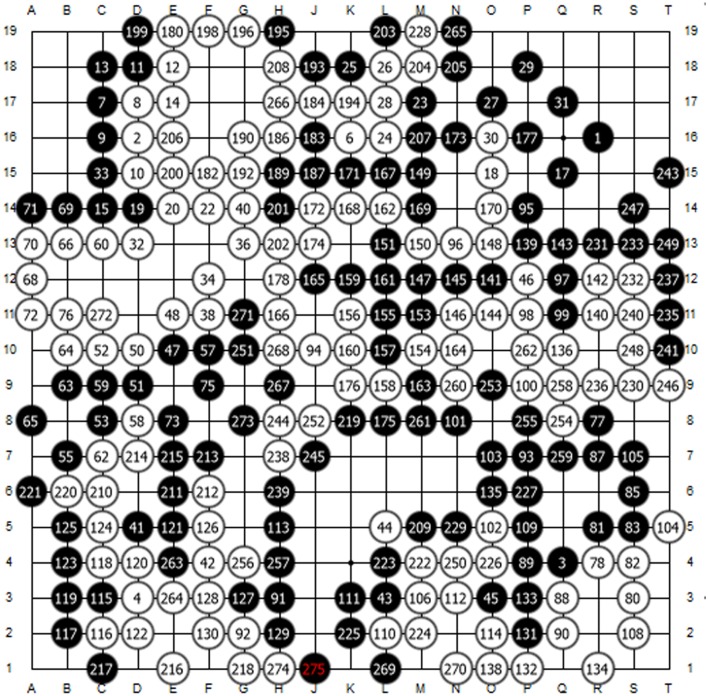
**GO game profile**.

To avoid the interference of the experiences for playing a GO game, none of them had ever played a GO game before the study. Before game training, all subjects from GO-game groups were invited to learn the rules for playing the game from websites, such as http://en.wikipedia.org/wiki/GO_(game). All participants were trained by a game player in the same club. After mastering the basic rules, each person from GO-game groups can finish the game as Figure 2 showed. In GO groups, two partners were combined randomly. If one patient could not find a partner, he or she watched the game played by other participants. Otherwise, he or she would play with one of GO-game staff.

### Neuropsychological tests

According to Diagnostic and Statistical Manual of Mental Disorders (DSM), all the cases were diagnosed by licensed neurological physicians including neurological examination, blood tests, and neuroimaging data (computed tomography or magnetic resonance imaging). Clinical Dementia Rating (CDR) scale is often used to detect memory and executive functions (Inoue et al., 2012). Furthermore, CDR is used not only to detect memory impairment but also to quantify dementia severity. CDR is also used to assess domains of cognition (memory, orientation and problem solving) and the domains of functions (community affairs, home, and hobbies and personal care) (Cedarbaum et al., 2013). Furthermore, the Mini Mental State Examination (MMSE) has been widely used for testing memory problems. Clinicians often diagnose dementia and assess its progression and severity by MMSE scores.

Depression is a frequent condition for AD patients, so the depressive severity was examined based on the Montgomery and Asberg Depression Rating Scale (MADRS) (Alonzo et al., 2013). There are 10 items for MADRS scoring from 0 to 60. The severities of depression were regarded as severe, moderate and mild based on different cut-off scores (Birnbaum et al., 2009). Furthermore, all participants was evaluated with the Kimberley Indigenous Cognitive Assessment of Depression (KICA-Dep) (Salvatore et al., 2013). There are 11 items for KICA-Dep, each of which is assigned as from “never” to “sometimes,” “a lot” and “all the time” based on a frequency scale.

Some AD patients suffer from anxiety disorder (Ramakers et al., 2013; Mormont et al., 2014) and anxiety was measured based on Hospital Anxiety and Depression Scale (HADS) (Hinz et al., 2014). The scores of HADS are presented as from 0 to 21, and higher scores stand for more severe anxiety. General functioning was evaluated by a Global Assessment of Functioning (GAF) scale (Mello et al., 2007). Life quality was assessed using RAND-36 (Mattila et al., 2012), which counts for wellbeing and functioning in eight dimensions. Alexithymia is a psychological problem and was evaluated using the Toronto Alexithymia Scale–20 (TAS-20) (Melin et al., 2010).

### ELISA analysis of BDNF

To determine serum levels of BDNF, 5 ml of blood samples were taken (8:00–11:00 a.m.) by venipuncture into a free-anticoagulant vacuum tube at before and after 6-month follow-up. The blood samples were centrifuged at 3000 g for 10 min, and serum was isolated and kept at −80°C until next step. The serum levels of BDNF were measured using a human BDNF ELISA kit (Adipo Bioscience, Santa Clara, CA, USA).

### Statistical analyses

All statistical analyses were performed with a SPSS 20.0 package (IBM China Company Limited, Beijing, China). The AD patients were compared by *t*-tests between GO groups and the control group without a GO intervention. One-Way ANOVA was used to explore the effect of a GO game on the levels of BDNF in AD patients. Spearman's rank correlation coefficient was conducted to identify the correlation between the depressive severity (Montgomery-Asberg Depression Rating Scale or RAND-36) and BDNF levels. There were statistically significant differences if *P* < 0.05.

## Results

### The baseline characters of AD patients

All 147 AD patients had medical histories consistent with AD and took neurological examinations (Supplemental Table 1). Controls were matched well to AD patients by gender and age at onset (*P* > 0.05). A previous report has found that BMI is associated with the risk of AD (Besser et al., 2014). To avoid the effects of BMI on present results, all patients were selected to guarantee that there were no statistically significant differences among three groups (*P* > 0.05) (Supplemental Table 1). Diabetes is an increasing epidemic and affects millions of the elderly worldwide. The chronic disease also affects brain function and contributes to an AD risk (Butterfield et al., 2014). Similarly, high blood pressure and coronary heart disease are also contributing factors for the development of AD (Qiu et al., 2006; Lattanzi et al., 2014). Furthermore, cigarette smoking has been reported to be also associated with incipient AD (Chang et al., 2012). Alcohol drinking can impair brain function, and cause dementia and geriatric cognitive disorders (Wiscott et al., 2001). The patients with long-period education have better cognitive functions than the AD patients with short-period education (Pradier et al., 2014). Furthermore, the anterior temporal lobes play an important role in melody recognition, and that music can significantly affect AD patients (Johnson et al., 2011). To avoid these interfering factors, all the elements were carefully examined to exclude their effects on final results (Supplemental Table 1). In neuropsychological tests, all AD patients had MMSE scores less than 26 and the CDR scale of 0.5 or over 0.5 (Supplemental Table 1). The results indicated that all AD subjects had clinical dementia. Similarly, all AD patients had the similar psychiatric test values and the prevalence of KICA-dep items in AD patients among LGGI, SGGI, and control groups (*P* > 0.05) (Supplemental Table 1 and Table 1). Therefore, the baseline characters of patients were basically similar among three groups.

**Table 1:** **The comparison for Prevalence of KICA-Dep items among AD patients**.

**KICA-Dep Items**	**Response**	**Control group**			**SGGI group**			**LGGI group**		
		**Baseline N(%)**	**6 months N(%)**	***P*-value**	**Baseline N(%)**	**6 months N(%)**	***P*-value**	**Baseline N(%)**	**6 months N(%)**	***P*-value**
Felt down, sad, no good	Never	5(10.2)	5(11.3)	0.587	5(10.2)	10(20.8)	0.001	5(10.2)	12(25.0)	0.001
	Sometimes	30(61.2)	26(59.1)	0.182	29(59.2)	30(62.5)	0.183	28(57.1)	31(64.6)	0.043
	A lot	5(10.2)	5(11.3)	0.210	5(10.2)	3(6.3)	0.002	6(12.2)	2(4.2)	0.001
	All the same	9(18.4)	8(18.2)	0.859	10(20.4)	5(10.4)	0.001	10(20.4)	2(4.2)	0.001
Felt like doing things that you usually like doing	Never	5(10.2)	5(11.4)	0.066	6(12.2)	4(8.3)	0.006	5(10.2)	4(8.3)	0.004
	Sometimes	25(51.0)	22(50.0)	0.393	25(51.0)	20(41.6)	0.002	25(51.0)	19(39.6)	0.002
	A lot	6(12.2)	5(11.4)	0.299	6(12.2)	7(14.6)	0.015	6(12.2)	8(16.7)	0.005
	All the same	13(26.5)	12(27.3)	0.180	12(24.5)	17(35.4)	0.001	13(26.5)	17(35.4)	0.001
Had trouble going to sleep, staying asleep, or sleeping too much	Never	5(10.2)	5(11.4)	0.804	5(10.2)	18(37.5)	0.001	5(10.2)	20(41.7)	0.001
	Sometimes	30(61.2)	26(59.1)	0.185	29(59.2)	20(41.7)	0.001	30(61.2)	18(37.5)	0.001
	A lot	4(8.2)	3(6.1)	0.119	4(8.2)	3(6.3)	0.048	5(10.2)	4(8.2)	0.008
	All the same	10(20.4)	6(22.7)	0.083	11(22.4)	7(14.6)	0.001	9(18.4)	6(12.5)	0.001
Felt tired or slack, and had no energy	Never	7(15)	10(20.0)	0.854	13(16.3)	21(27.5)	0.085	13(16.3)	21(27.5)	0.085
	Sometimes	24(50)	28(57.5)	0.341	39(48.7)	37(49.3)	0.874	39(48.7)	37(49.3)	0.874
	A lot	12(25)	8(17.5)	0.246	19(23.8)	14(18.8)	0.440	19(23.8)	14(18.8)	0.440
	All the same	6(10)	2(5.0)	0.230	9(11.2)	3(3.8)	0.072	9(11.2)	3(3.8)	0.072
Eating too much or eating only a little bit.	Never	5(10.2)	4(9.1)	0.197	5(10.2)	15(31.3)	0.001	5(10.2)	16(33.3)	0.001
	Sometimes	4(8.2)	4(9.1)	0.133	5(10.2)	25(52.1)	0.001	4(8.2)	25(52.1)	0.001
	A lot	16(32.7)	14(31.8)	0.210	15(30.6)	4(8.3)	0.001	16(32.7)	4(8.3)	0.001
	All the same	24(49)	22(50)	0.512	24(49)	4(8.3)	0.001	24(49)	3(6.3)	0.001
Felt bad or shamed that you let yourself or your family down.	Never	8(16.3)	7(15.9)	0.514	7(14.3)	17(35.4)	0.001	7(14.3)	18(37.5)	0.004
	Sometimes	4(8.2)	4(9.1)	0.614	5(10.2)	15(31.3)	0.001	6(12.2)	16(33.3)	0.001
	A lot	17(34.7)	15(34.1)	0.693	17(34.7)	8(16.7)	0.001	16(32.7)	7(14.6)	0.001
	All the same	20(40.8)	18(40.9)	0.875	20(40.8)	8(16.7)	0.001	20(40.8)	7(14.6)	0.001
Had trouble paying attention or concentrating on things	Never	6(12.2)	5(11.4)	0.449	7(14.3)	16(33.3)	0.001	7(14.3)	17(35.4)	0.001
	Sometimes	10(20.4)	9(20.5)	0.526	11(22.4)	21(43.8)	0.001	11(22.4)	22(45.8)	0.001
	A lot	14(28.6)	13(29.5)	0.514	13(26.5)	6(12.5)	0.001	14(28.6)	4(8.3)	0.001
	All the same	19(38.8)	17(38.6)	0.948	18(36.7)	5(10.4)	0.001	17(34.7)	5(10.4)	0.001
Been told that you are speaking or moving too slowly or fast	Never	5(10.2)	4(9.1)	0.194	5(10.2)	15(31.3)	0.001	5(10.2)	15(31.3)	0.001
	Sometimes	6(12.2)	5(11.4)	0.445	6(12.2)	18(37.5)	0.001	6(12.2)	18(37.5)	0.001
	A lot	12(24.5)	11(25)	0.551	12(24.5)	7(14.6)	0.001	13(26.5)	8(16.7)	0.001
	All the same	26(53.1)	24(54.5)	0.303	26(53.1)	8(16.7)	0.001	25(51)	7(14.6)	0.001
Had thoughts that you would be better off dead	Never	3(6.1)	3(6.8)	0.204	4(8.2)	17(35.4)	0.001	4(8.2)	18(37.5)	0.001
	Sometimes	6(12.2)	5(11.4)	0.752	6(12.2)	16(33.3)	0.001	6(12.2)	17(35.4)	0.001
	A lot	12(24.5)	11(25)	0.822	11(22.4)	9(18.8)	0.001	11(22.4)	7(14.6)	0.001
	All the same	28(57.1)	25(56.8)	0.730	28(57.1)	6(12.5)	0.001	28(57.1)	6(12.5)	0.001
Thought of hurting yourself	Never	4(8.2)	4(9.1)	0.105	4(8.2)	14(29.2)	0.001	4(8.2)	15(31.3)	0.001
	Sometimes	4(8.2)	4(9.1)	0.138	4(8.2)	19(39.6)	0.001	4(8.2)	19(39.6)	0.001
	A lot	14(28.6)	12(27.3)	0.357	15(30.6)	7(14.6)	0.001	15(30.6)	7(14.6)	0.001
	All the same	27(55.1)	24(54.5)	0.548	26(53.1)	8(16.7)	0.001	26(53.1)	7(14.6)	0.001
Felt angry	Never	2(4.1)	2(4.5)	0.627	2(4.1)	18(37.5)	0.001	3(6.1)	17(35.4)	0.001
	Sometimes	3(6.1)	3(6.8)	0.322	3(6.1)	17(35.4)	0.001	2(4.1)	18(37.5)	0.001
	A lot	24(49)	22(50)	0.512	23(46.9)	7(14.6)	0.001	23(46.9)	6(12.5)	0.001
	All the same	20(40.8)	17(38.6)	0.142	21(42.9)	6(12.5)	0.001	21(42.9)	7(14.6)	0.001

*n = 49 in each group*.

### Statistical analyses for the outcomes at 6-month follow-up

Statistical analyses showed that a GO-game intervention significantly decreased the mean score of MADRS of 4.72 (95% CI, 0.69–9.12) compared with those in a control group after 6-month follow-up (*P* < 0.05) (Supplemental Table 2). In the similar way, a GO-game intervention also reduced mean score of HADS of 1.75 (95% CI, 0.17–3.68) when compared with those from controls (*P* < 0.05) (Supplemental Table 2). In contrast, a GO-game intervention significantly increased the mean score of GAF of 4.95 (95% CI, −1.37–9.18) and RAND-36 of 4.61 (95% CI, −2.75–11.32) when compared with a control group (*P* < 0.05) (Supplemental Table 2). For TAS-20, there was no statistically significant difference in three groups (*P* > 0.05) (Supplemental Table 2). The results may be caused by alexithymic personality traits, which are closely related with gender, advanced age, educational level and social intelligence (Koelkebeck et al., 2010).

### A GO game improves the symptoms of AD

The analysis for KICA-Dep items showed that GO game relieved the symptoms of AD patients compared with those from a control group (Table 1). In the 11 items of KICA-Dep, a control group could not attenuate the symptoms of AD patients and there was no statistically significant difference for most items (*P* > 0.05) except of two ones (Table 1). Comparatively, a GO game ameliorated the symptoms of depression and there were statistically significant differences for most items (*P* < 0.05) except of two items (Table 1). After a 6-month GO-game intervention, the severity of AD was decreased significantly (*P* < 0.05) (Table 1).

### The levels of BDNF are negatively related with stratification of CDR

When AD severity was stratified by CDR scales at onset (χ^2^ = 6.8, *P* = 0.03), significant differences of BDNF levels were observed in lower (CDR 0.5), middle (CDR 1), and higher CDR (CDR 2^+^). There were statistically significant differences for BDNF levels between CDR 0.5 and 2^+^ (*P* < 0.05). BDNF levels were negatively related with CDR scales, suggested that low levels of BDNF were related with AD risk since the BDNF levels were higher in lower CDR scores compared to those in higher CDR scores (χ^2^ = 214, *P* < 0.01) while CDR scores have been used to evaluate the severity of AD (Clark et al., 2014).

### Association between an AD episode and the protein levels of BDNF

Elevated levels of BDNF were related with a decrease in MADRS scores and an increase in RAND-36 scales (*P* < 0.01). There was a strong negative relationship between the severity of AD and the levels of BDNF because the rho values were less than −0.5 based on the calculation of Spearman's rank correlation coefficient.

### A GO game enhances the serum protein levels of BDNF in AD patients

**Figure 3:**
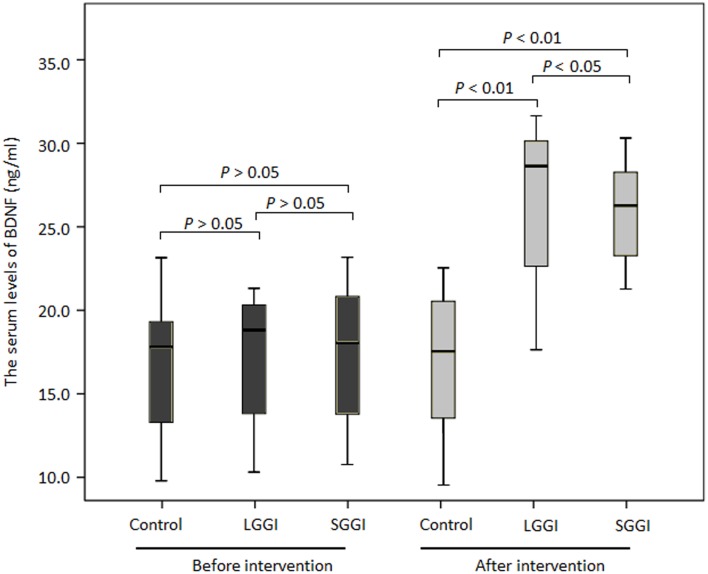
**Serum BDNF levels in control, LGGI and SGGI groups before and after 6-month follow-up**. Comparative analyses were carried out using ANOVA followed by a Duncan test. Values were expressed as mean ± SD. *N* = 49 cases in each group. The bars in the boxes were average expression and the boxes represented 95% of the samples. The error bars were above or below the boxes.

The study consisted of 147 AD patients before and after 6-month follow-up. The socio-demographic was list in Supplemental Table 1 and balanced based on age, gender, educational levels, life habits and medicine therapy. The results showed that BDNF levels were similar in three groups before GO game intervention. After 6-month intervention, serum BDNF levels were significantly higher in subjects receiving SGGI and LGGI (respectively, 24.02 ± 7.16 and 28.88 ± 4.12 ng/ml, *t* = 0.345, *P* = 0.051) when compared with controls (17.28 ± 7.75 ng/ml) (*t* = 3.423, *P* = 0.001) (Figure 3). All these results implied that a GO game promotes the production of BDNF.

## Discussion

A GO game is very popular in China, Japan, and Korean, and can be widely played. More importantly, a GO game has been proved to be very interesting for its rich strategies with simple rules and attracts many elderly adults. The game is more suitable to be developed to prevent the progression of AD. We investigated the effects of the game on AD and related molecular mechanisms. On the other hand, physical activities can certainly delay the loss of autonomy in AD patients and are also useful strategies for delaying the complications of AD (Rolland et al., 2000). During the recruitment, some AD patients like physical exercises but they dislike playing GO game. In the similar cases, some AD patients like playing the game but they dislike physical exercises. Only a few AD patients like playing a GO game and physical exercises. The sample size will be a problem for considering both physical exercises and a GO game. Furthermore, the effects of physical exercises on AD patients have been reported. Thus, we only consider the effects of a GO game on AD here. The patients will be excluded if they dislike playing the game. Importantly, a GO game showed effective anti-depression results with few side effects. Meanwhile, we interpreted the possible outcome by using GO-game-specific and computational methods with a particular motivation to detect therapeutically-relevant phenomena.

The strength of the paper is its technique in clinical practice. Furthermore, each person in GO groups could play the game well with basic rules. On the other hand, the combination of various techniques was utilized. Brain imaging and self-ratings, computational game analyses and psychiatric assessment was undertaken, resulting in complementary and myriad measures access to various underlying determinants of an intervention. Some brain imaging techniques, such as fMRI and PET, can provide distinct spatial information, but which cannot provide time and event-related correlations. Depression has been reported to be correlated with a hypo-activation of left brain activity and a hypo-inactivation of right brain activity (Richieri et al., 2012), which can be ameliorated with the time going after playing the game.

MADRS is one of the most effective tools for detecting the depression in AD patients and higher MADRS score suggests severe depression (Müller-Thomsen et al., 2005). We used the MADRS to measure the changes of depression severity. The results showed that a GO game significantly decreased mean score of MADRS of 4.72 (95% CI, 0.69–9.12) when compared with controls after 6-month follow-up (*P* < 0.05, Supplemental Table 2). HADS is also an important tool to detect the depression of AD (Pietrzak et al., 2015), so we also measured the HADS scores here. In the similar results, a GO game decreased the mean score of HADS of 1.75 (95% CI, 0.17–3.68) when compared with controls in the same period (*P* < 0.05, Supplemental Table 2). Furthermore, KICA-Dep has been reported to very useful for detecting the depressive symptoms in AD patients. The results indicated that a GO game improved 9 of 11 items of KICA-Dep compared with controls after 6-month follow-up (Table 1). On the other hand, GAF has been used to evaluate the overall improved quality of life in AD patients (Lu et al., 2006). Thus, we measured GAF and found that a GO game increased the mean score of GAF of 4.95 (95% CI, −1.37–9.18) and RAND-36 of 4.61 (95% CI, −2.75–11.32) when compared with those in a control group after 6-month follow-up (*P* < 0.05, Supplemental Table 2).

Although a GO game showed effective results for ameliorating the symptoms of AD, the molecular mechanism remains unknown. Previous work demonstrates that AD patients have reduced levels of BDNF in the brain and serum. Additionally, animal-based studies also showed protective effects of BDNF against Amyloid beta-protein (Abeta)-induced neurotoxicity (Psotta et al., 2015). Abeta1-42 exhibits neurotoxicity and induces neural death. Abeta1-40 can inhibit the fibril formation of Abeta1-42 (Zou et al., 2003). In contrast, Abeta1-16, Abeta25-35, and Abeta40-1 prevent neither the fibril formation of Abeta1-42 nor Abeta1-42-induced neural death. Here, we measured serum levels of BDNF in AD patients and found that a GO game increased serum BDNF levels in SGGI and LGGI groups (24.02 ± 7.16 and 28.88 ± 4.12 ng/ml respectively, *t* = 0.345, *P* = 0.051) compared those in controls (17.28 ± 7.75 ng/ml) (*t* = 3.423, *P* = 0.001) after 6-month follow-up. Furthermore, the serum levels of BDNF showed a negative relation with MADRS and a positive relation with RAND-36 scores (*P* < 0.01). A GO game ameliorates AD patients by up-regulating BDNF levels. Thus, A GO game is effective to prevent the development of AD.

Alexithymia is considered for its defects in regulating feelings (Goerlich et al., 2011) and typically co-occurs with depression (Gilbert et al., 2014). However, alexithymia is a complex brain disorder with a cluster of deficits in the recognition (Reker et al., 2010). Here we found that a GO game was not an effective way for the treatment of alexithymia compared with that from a control group (*P* > 0.05) (Table 1). In any way, a GO game can improve the life quality in other aspects (*P* < 0.05) (Supplemental Table 2).

In addition, evoking and dealing with emotions is usually related with a GO game, which fit the treatment of emotional disorders just like depression. Although all AD patients wanted to learn and play a GO game before the study, someone kept active exercises and a few kept passive exercises for the game with time going. We found that most winners were active for playing the game while a few losers were passive for playing the game. We would let the game trainers play with these passive losers and let them win on purpose. Most of them could become active again for playing the game finally. In order to gain an insight into the effectiveness of the game, we also aimed to develop novel analytical methods for clinical purposes, such as kinematics, electromyographic and electroencephalographic (EEG) data (Barlaam et al., 2011). The new concept is also needed to understand the mechanisms by studying symptoms, behaviors, or biomarkers, which are different to traditional classification for the mental disorders (Badcock and Hugdahl, 2014). Meanwhile, the study will provide valuable information for the therapy of depression.

## Limitations

A GO game affects the progression of depression but the molecular mechanism remains unknown. Much evidence indicates that chronic stress and low levels of BNDF are the key causes of depression (Banerjee et al., 2014; Vásquez et al., 2014). However, the clinical relevance of a GO game intervention and the levels of BDNF remain unknown. Here, we firstly reported that a GO game ameliorated the depression by affecting the serum levels of BDNF. We found the relationships between SGGI or LGGI and levels of BDNF. The results showed that lower concentration of BDNF might be specific for the depressive state in AD patients. BDNF is a potential biomarker for adjuvant diagnosis and therapy of an AD episode. However, further studies are necessary to define a functional role of BDNF in preventing the development of depression.

Certainly, the present trial still has its limitations in a statistical sense. The sample size was sufficient to detect an effect in the primary outcome at 6-months, but not at longer period although we found the effect tended to persist. Second, the present trial was the GO game intervention. It remained unknown whether a long-term GO game was better than a short-term GO game. At least, there are no reports showing such indication yet. However, we still believe that a GO game intervention may be more suitable for a few populations only received medicine therapy.

Finally, a major problem with the design is that the control group did not receive any treatment. Therefore, it is not possible to know whether the changes reported are because the treatment groups received attention from and interacted with colleagues and staff or because of the game itself. A “placebo” procedure will be necessary to enable solid conclusions. Unfortunately, the “placebo” group is hard to be designed because no game can be matched GO game well according our experiences. Being interesting is very important to play a game. Gomoku, also called Five in a Row, can be played on a GO board. From May 6th, 2012 to August 12th, 2013, a total of 50 AD patients were recruited. All the participants did not have the experiences of playing Gomoku and showed interests for the game before the study. However, more than 50% patients lost their passions for the game after 2-month follow-up and the work was unavoidably stopped. Fortunately, less than 10% patients lost enthusiasm for GO game even after 6-month. Furthermore, these patients still could show enthusiasm again for GO game when they won in subsequent matches, which were designed on purpose by the GO game staff. We think that a GO game shows its beneficial functions on AD patients since the enthusiasm still can be maintained after 6-month follow-up.

## Clinical and mechanistic implications

A GO game can improve the life quality of AD patients. None complained of playing a GO game and no routine neurological exam could identify any adverse effect. Playing a GO game should be developed a public activity since the game intervention can reduce the symptoms of AD. Its mechanistic implication may be that many strategies involve in the play and the number of possible games is vast (there are about 10^120^ possible results for playing the game), although the rules of game are very simple. Given the substantial changes in the game, the game attracts an ever-growing older population. Playing a simple and inexpensive game can improve the cognitive functions of AD patients. Millions of AD patients are looking for a technologically-oriented game to enhance their cognitive skills, which can prevent the development of AD.

In sum, a GO game is feasible and effective to improve life quality of AD patients by reducing their depression. A GO game intervention reduces AD severity by increasing the level of BDNF. Thus, a GO game should be developed as a new method for the therapy of AD.

## Conflict of interest statement

The authors declare that the research was conducted in the absence of any commercial or financial relationships that could be construed as a potential conflict of interest.
