# Supplementary material for: The impacts of a GO-game (Chinese chess) intervention on Alzheimer disease in a Northeast Chinese population
Source: Front Aging Neurosci. 2015 Aug 25;7:163. doi: 10.3389/fnagi.2015.00163 (PMC4548213; doi:10.3389/fnagi.2015.00163)
Supplement: Supplementary file 1 [file Table1.DOC]

**Table 1** Baseline characteristics of AD patients

|  | Control (n = 49) | LGGI (n = 49) | SGGI (n = 49) | Mean difference (95% CI) | Odds ratio  (95% CI) | P value |
| --- | --- | --- | --- | --- | --- | --- |
| Age: years, mean(s.d.) | 41.9  (11.8) | 42.1  (12.2) | 42.5  (12.5) | 0.58 (-5.60 to 4.89) |  | 0.90 |
| Male, n (%) | 21 (42.9) | 20(40.8) | 22(44.9) |  | 1.45 (0.28 to 5.27) | 0.84 |
| Smoker/Non-smoker | 24/25 | 25/24 | 25/24 |  | 1.29 (0.31 to 2.98) | 0.47 |
| Drinker/Non-drinker | 23/26 | 21/28 | 21/28 |  | 1.63 (0.26 to 3.88) | 0.66 |
| Renal failure (%) | 33 (67.3) | 34 (69.4) | 35 (71.4) |  | 1.25 (0.37 to 3.29) | 0.59 |
| Diabetes (%) | 27 (55.2) | 26 (53.1) | 25 (51.0) |  | 1.34 (0.54 to 5.17) | 0.48 |
| Education levels (> 9 years), n (%) | 11(22.4) | 13 (26.5) | 12 (24.5) |  | 1.39 (0.43 to 4.86) | 0.53 |
| Spouse, n (%) | 42 (85.7) | 40 (81.6) | 41 (83.7) |  | 1.31 (0.25 to 3.77) | 0.64 |
| Career (working overtime), n (%) | 31 (63.3) | 30 (61.2) | 32 (65.3) |  | 1.42 (0.41 to 5.24) | 0.52 |
| Months of hemodialysis , n (s.d) | 26 (13) | 27 (12) | 28 (14) |  | 1.21 (0.50 to 5.57) | 0.38 |
| Anxiety (cut-off score 8 in Hospital Anxiety and Depression Scale), n (%) | 42 (85.7) | 43 (87.8) | 44 (89.7) |  | 1.51(1.43 to 1.59) | 0.51 |
| Musical background, n (%) |  |  |  |  |  |  |
| Sings | 9 (18.3) | 8 (16.3) | 8 (16.3) |  | 1.24 (0.33 to 3.87) | 0.65 |
| Plays an instrument | 9 (18.3) | 10 (20.4) | 9 (18.3) |  | 1.29 (0.32 to 4.17) | 0.31 |
| Has musical training | 5(10.2) | 6 (12.2) | 5 (10.2) |  | 1.16 (0.66 to 2.94) | 0.43 |
| a musician/singer | 4(8.2) | 5 (10.2) | 4 (8.2) |  | 1.55 (0.49 to 4.17) | 0.28 |
| Current medication, n (%) |  |  |  |  |  |  |
| Antidepressant medication | 17 (34.7) | 18 (36.7) | 18 (36.7) |  | 0.83 (0.51 to 2.39) | 0.54 |
| Selective serotonin reuptake inhibitors | 21 (42.9) | 22(44.9) | 23(46.9) |  | 1.25 (0.35 to 3.44) | 0.32 |
| Serotonin and noradrenaline reuptake inhibitors | 7 (14.3) | 6(12.4) | 7(14.3) |  | 1.42 (0.53 to 3.85) | 0.85 |
| Psychiatric test scores, mean (s.d.) |  |  |  |  |  |  |
| Montgomery-Asberg Depression Rating Scale | 23.9 (7.8) | 24.1 (7.1) | 24.6 (7.4) | 1.59 (75.1 to 1.87) |  | 0.33 |
| Anxiety score (Hospital Anxiety and Depression Scale) | 12.6 (4.5) | 11.2 (4.0) | 11.3 (4.1) | 1.02 (74.6 to 0.81) |  | 0.27 |
| Global Assessment of Functioning score | 59.4 (6.8) | 58.9 (8.5) | 58.1 (8.3) | 1.12 (73.4 to 3.2) |  | 0.65 |
| Toronto Alexithymia Scale - 20 | 54.6(13.8) | 52.6 (13.1) | 53.7 (14.3) | 1.51 (77.2 to 5.4) |  | 0.71 |
| RAND-36 score | 52.3 (16.1) | 53.1 (15.2) | 53.6 (15.9) | 2.24 (75.6 to 7.8) |  | 0.60 |
| MMSE scores | 19.8 ± 4.9 | 19.1 ± 4.7 | 18.8 ± 4.3 | 4.5 (24.3 to 14.0) |  | 0.55 |
| **CDR scale** |  |  |  |  |  |  |
| CDR 0 (Cases) | 0 | 0 | 0 |  |  |  |
| CDR 0.5(Cases) | 29 | 28 | 27 |  |  |  |
| CDR 1(Cases) | 10 | 12 | 14 |  |  |  |
| CDR 2+(Cases) | 10 | 9 | 8 |  |  |  |
