# Supplementary material for: The impacts of a GO-game (Chinese chess) intervention on Alzheimer disease in a Northeast Chinese population
Source: Front Aging Neurosci. 2015 Aug 25;7:163. doi: 10.3389/fnagi.2015.00163 (PMC4548213; doi:10.3389/fnagi.2015.00163)
Supplement: Supplementary file 2 [file Table2.DOC]

**Table 2** Statistical analyses for six-month follow-up outcomes

|  | 6-month follow-up, (n =147)^a^ | | | | |
| --- | --- | --- | --- | --- | --- |
| Outcome | Mean  (s. d.) | Change from baseline (s. d.) | Mean difference (95% CI) | T-test | P |
| Montgomery-Asberg Depression Rating Scale |  |  |  |  |  |
| Control group | 16.73 (9.46) | -6.24 | 4.72 (0.69 to 9.12) | 2.31 | 0.02 |
| SGGI group | 13.32 (8.25) | -11.36 |
| LGGI group | 12.10 (8.25) | -12.13 |
| Hospital Anxiety and Depression Scale - Anxiety |  |  |  |  |  |
| Control group | 8.07（4.26） | -2.08 | 1.75 (0.17 to 3.68) | 2.22 | 0.03 |
| SGGI group | 6.59 (4.11) | -3.95 |
| LGGI group | 5.89 (3.34) | -4.12 |
| Global Assessment of Functioning |  |  |  |  |  |
| Control group | 63.56(9.74) | 6.02 | -4.95 (-9.18 to 1.37) | -2.17 | 0.03 |
| SGGI group | 69.48 (9.16) | 11.42 |
| LGGI group | 71.55 (11.21) | 12.28 |
| Toronto Alexithymia Scale - 20 |  |  |  |  |  |
| Control group | 48.53(12.31) | -4.32 | 2.17 (-2.58 to 6.59) | 0.96 | 0.46 |
| SGGI group | 46.24 (11.99) | -5.87 |
| LGGI group | 47.15 (12.32) | -6.05 |
| RAND-36 |  |  |  |  |  |
| Control group | 61.34 (17.54) | 9.12 | -4.61 (-11.32 to 2.75) | -2.39 | 0.04 |
| SGGI group | 66.42 (19.83) | 14.21 |
| LGGI group | 68.22 (21.11) | 15.18 |

^a^Control group n = 49; LGGI and SGGI groups, n = 49 respectively.
